# The Epichaperome Matrix Theory: A systems-level model of active molecular organization

**DOI:** 10.1016/j.cstres.2026.100210

**Published:** 2026-09-01

**Authors:** Maxim Shevtsov

**Affiliations:** Department of Radiation Oncology, Klinikum rechts der Isar, Technical University of Munich, Ismaninger Str. 22, Munich 81675, Germany

**Keywords:** Epichaperome, Molecular chaperones, Heat shock proteins, Stress adaptation, Systems biology, Molecular transport

## Abstract

**Background:**

Epichaperomes are stable, stress-induced supramolecular assemblies formed through extensive integration of molecular chaperones, co-chaperones, signaling proteins, and client proteins. Although increasing evidence indicates that epichaperomes act as organizational hubs that coordinate proteostasis and signaling networks in cancer, neurodegenerative disorders, and chronic inflammatory diseases, their potential physical roles in cellular organization remain largely unexplored.

**Results:**

Here, the Epichaperome Matrix Theory, a systems-level theoretical framework that conceptualizes the epichaperome as a dynamic, nonequilibrium biomolecular matrix possessing emergent transport-regulatory properties, is proposed. In this model, epichaperome assemblies generate heterogeneous electrostatic landscapes through the collective distribution of charged amino acid residues, phosphorylation-dependent charge accumulation, ATP-driven conformational dynamics, and high-order network connectivity. By integrating principles from Poisson-Boltzmann electrostatics, Nernst-Planck transport theory, active matter physics, percolation theory, graph theory, biomolecular condensate thermodynamics, and porous hydrogel transport models, a mathematical description in which epichaperomes function as adaptive organizational scaffolds capable of influencing molecular flux, signaling efficiency, and spatial coordination within cells is developed. This framework is further extended through the Transcellular Epichaperome Continuum Hypothesis, proposing that intracellular epichaperomes may be functionally coupled to plasma membrane-associated and extracellular epichaperome assemblies, forming a multiscale organizational network spanning individual cells, tissues, and organ systems. In this extended model, membrane-bound epichaperomes act as coupling interfaces between intracellular and extracellular compartments, while secreted chaperones, extracellular vesicles, and extracellular protein assemblies contribute to intercellular connectivity. Mathematical analysis predicts the emergence of percolating transport networks, electrostatic coupling domains, synchronized conformational dynamics, and stress-responsive communication pathways when epichaperome connectivity exceeds critical thresholds.

**Conclusions:**

The proposed framework suggests that epichaperomes may represent more than stress-associated protein interaction networks and could function as dynamic organizational matrices integrating molecular organization, signaling, and adaptive responses across multiple biological scales. Although the theory remains speculative and currently lacks direct experimental validation, it generates testable predictions regarding membrane-associated epichaperomes, extracellular epichaperome assemblies, electrostatic organization, and intercellular transport behaviors. By providing a unified theoretical foundation linking stress biology, chaperone networks, systems biology, and biophysics, this work expands the conceptual landscape of epichaperome research and identifies new directions for investigating the role of higher-order chaperome organization in health and disease.

## Background

Cellular survival depends on the capacity of biological systems to maintain molecular organization despite constant exposure to environmental, metabolic, and pathological stress. To preserve proteome integrity, cells have evolved an extensive network of molecular chaperones and co-chaperones collectively known as the chaperome.^1^, ^2^ Traditionally, molecular chaperones have been viewed primarily as protein quality-control factors that facilitate protein folding, prevent aggregation, promote refolding of damaged proteins, and coordinate proteostasis under both physiological and stress conditions.^3^ Among these, heat shock proteins (HSPs), including HSP90, HSP70, HSP110, HSP60, and their numerous co-chaperones, occupy central positions in the regulation of cellular homeostasis and adaptive stress responses.^4^

Over the past 2 decades, advances in proteomics, systems biology, and network analysis have revealed that chaperones do not operate as isolated molecular machines. Instead, they participate in highly interconnected protein interaction networks that dynamically reorganize in response to cellular demands.^5^ Under normal conditions, chaperone interactions are generally transient and highly regulated, allowing rapid adaptation to changing intracellular environments. However, under conditions of chronic stress, including cancer, neurodegenerative disorders, metabolic disease, and persistent inflammation, chaperome architecture undergoes profound remodeling. Increasing evidence indicates that molecular chaperones can assemble into stable, highly interconnected supramolecular structures termed epichaperomes.^6^, ^7^, ^8^ Epichaperomes exhibit prolonged stability, increased network connectivity, and extensive integration of signaling, metabolic, and structural proteins.

The concept of the epichaperome has fundamentally altered our understanding of stress biology.^6^, ^7^, ^9^ Rather than functioning solely as protein-folding machinery, epichaperomes appear to operate as organizational hubs capable of coordinating multiple cellular processes simultaneously. Studies in cancer cells have demonstrated that epichaperomes integrate signaling pathways, stabilize oncogenic networks, and influence the functional connectivity of proteins across large portions of the proteome.^10^ Similar structures have been identified in neurodegenerative diseases, where they contribute to pathological protein interactions and network dysfunction.^11^ These observations suggest that epichaperomes represent emergent stress-induced systems that extend beyond conventional definitions of molecular chaperones.

Recently our group introduced the concept of a plasma membrane-bound epichaperome, a specialized stress-induced supramolecular assembly of HSP90, HSP70, co-chaperones, signaling proteins, and client proteins associated with the plasma membrane and membrane lipid rafts.^12^, ^13^ In contrast to the cytosolic epichaperome, which primarily organizes intracellular proteostasis, signaling, and stress adaptation within the cytoplasmic milieu, the membrane-bound epichaperome is hypothesized to function as an interface linking intracellular chaperome networks with membrane receptors, lipid rafts, cytoskeletal elements, extracellular vesicles, and extracellular chaperone assemblies. Consequently, the plasma membrane-bound epichaperome may possess distinct organizational and signaling properties, enabling transmembrane coordination of molecular transport, mechanochemical signaling, and intercellular communication beyond the classical functions attributed to cytosolic epichaperomes.

Despite substantial advances in understanding epichaperome composition and biological significance, the physical principles governing their large-scale organization remain poorly understood. Existing experimental studies have characterized epichaperomes primarily as stable, highly interconnected chaperone-protein interaction networks that undergo dynamic remodeling in response to cellular stress and disease.^7^, ^14^ Such representations successfully capture connectivity patterns but often fail to explain how these assemblies influence intracellular spatial organization, molecular trafficking, signaling efficiency, and systems-level coordination. Living cells are not simply collections of interacting proteins; they are highly organized nonequilibrium systems in which information, metabolites, ions, and macromolecules must be transported efficiently across complex intracellular environments. The possibility that epichaperomes contribute directly to such organizational processes has received comparatively little theoretical attention.

Recent developments in cell biology increasingly emphasize the importance of mesoscale organization.^15^ Biomolecular condensates, phase-separated compartments, cytoskeletal networks, membrane nanodomains, and intracellular electrostatic landscapes have emerged as fundamental regulators of cellular architecture.^16^, ^17^, ^18^ These systems generate spatial heterogeneity that guides molecular movement, concentrates signaling components, and enhances reaction efficiency. Importantly, many of these organizational principles arise from collective properties of large molecular assemblies rather than from the activities of individual proteins. Such observations raise an intriguing possibility: could epichaperomes similarly possess emergent physical properties that influence molecular transport and information flow throughout the cell?

The present work explores this possibility by proposing the *Epichaperome Matrix Theory* (EMT), a systems-level framework that conceptualizes the epichaperome as a dynamic, electrically polarized, nonequilibrium biomolecular scaffold. In this model, the epichaperome is viewed not merely as a protein interaction network but as a three-dimensional matrix capable of generating spatial electrostatic asymmetry through the collective organization of charged proteins, post-translational modifications, ATP-dependent conformational dynamics, and network connectivity. The resulting electrostatic landscape is hypothesized to create preferential pathways that influence the movement of ions, metabolites, proteins, nucleic acids, extracellular vesicles, and signaling molecules.

The theoretical basis for this framework emerges from several established biological observations. First, proteins comprising epichaperomes contain extensive distributions of charged amino acid residues that generate localized electrostatic fields. Second, phosphorylation and other post-translational modifications substantially alter molecular charge distributions within signaling networks.^14^ Third, ATP-driven conformational cycling continuously reorganizes molecular interactions and structural connectivity. Fourth, epichaperomes display characteristics reminiscent of biomolecular condensates and phase-organized systems capable of generating mesoscale order.^5^, ^19^ Together, these features suggest that epichaperomes may possess physical properties extending beyond simple molecular connectivity.

It is important to clarify that the EMT is a theory-driven mathematical framework rather than a data-generated or experimentally fitted model. It formalizes biologically motivated assumptions and established physical principles into a system of mathematical relationships and constraints. The equations therefore represent consequences of the proposed theoretical framework rather than estimates derived from experimental datasets. The model is intended to generate testable predictions and identify relationships among epichaperome organization, electrostatic properties, molecular transport, and network dynamics. Its validation will require future comparison of these predictions with independently obtained experimental evidence across appropriate biological systems.

To explore these ideas quantitatively, the present theory integrates concepts from multiple disciplines, including membrane electrostatics, active matter physics, reaction-diffusion systems, electrophoretic transport, percolation theory, biomolecular condensate thermodynamics, and porous charged hydrogel mechanics. Electrostatic potentials generated by epichaperome assemblies are described using Poisson-Boltzmann formalism, while molecular movement is modeled through Nernst-Planck transport equations coupled to ATP-dependent conformational dynamics. Network connectivity is represented using graph-theoretical approaches, and phase behavior is incorporated through free-energy formulations commonly applied to nonequilibrium biological systems. By combining these theoretical frameworks, the model provides a unified description of how structural organization, electrostatic asymmetry, and dynamic remodeling could collectively influence intracellular transport processes.

The EMT should be viewed as a conceptual and mathematical framework rather than a description of established biological reality. Its purpose is not to replace current understanding of membrane transport, diffusion, cytoskeletal trafficking, or circulatory systems. Instead, it seeks to explore whether epichaperomes may constitute an additional organizational layer superimposed upon these classical mechanisms, enhancing molecular coordination through emergent collective properties.

Such a perspective is consistent with broader trends in contemporary systems biology, where increasing attention is being directed toward understanding how higher-order molecular assemblies generate behaviors that cannot be predicted from individual molecular components alone. Complex biological systems frequently exhibit emergent phenomena, including self-organization, collective dynamics, pattern formation, and adaptive responses. Epichaperomes, by virtue of their extensive connectivity and stress-dependent assembly, may represent an important example of such emergent organization. Investigating their potential physical functions may therefore provide new insights into cellular adaptation, disease progression, and the integration of signaling networks across multiple spatial scales.

The objective of this work is to establish a theoretical foundation for examining the possibility that epichaperomes function as electroactive organizational matrices capable of influencing molecular transport and communication. By integrating principles from stress biology, biophysics, and systems theory, the proposed framework aims to stimulate experimental investigation into previously unexplored dimensions of epichaperome function and to provide a basis for future quantitative studies of stress-induced molecular organization in health and disease.

## Epichaperome Matrix Theory

**Fig. 1:**
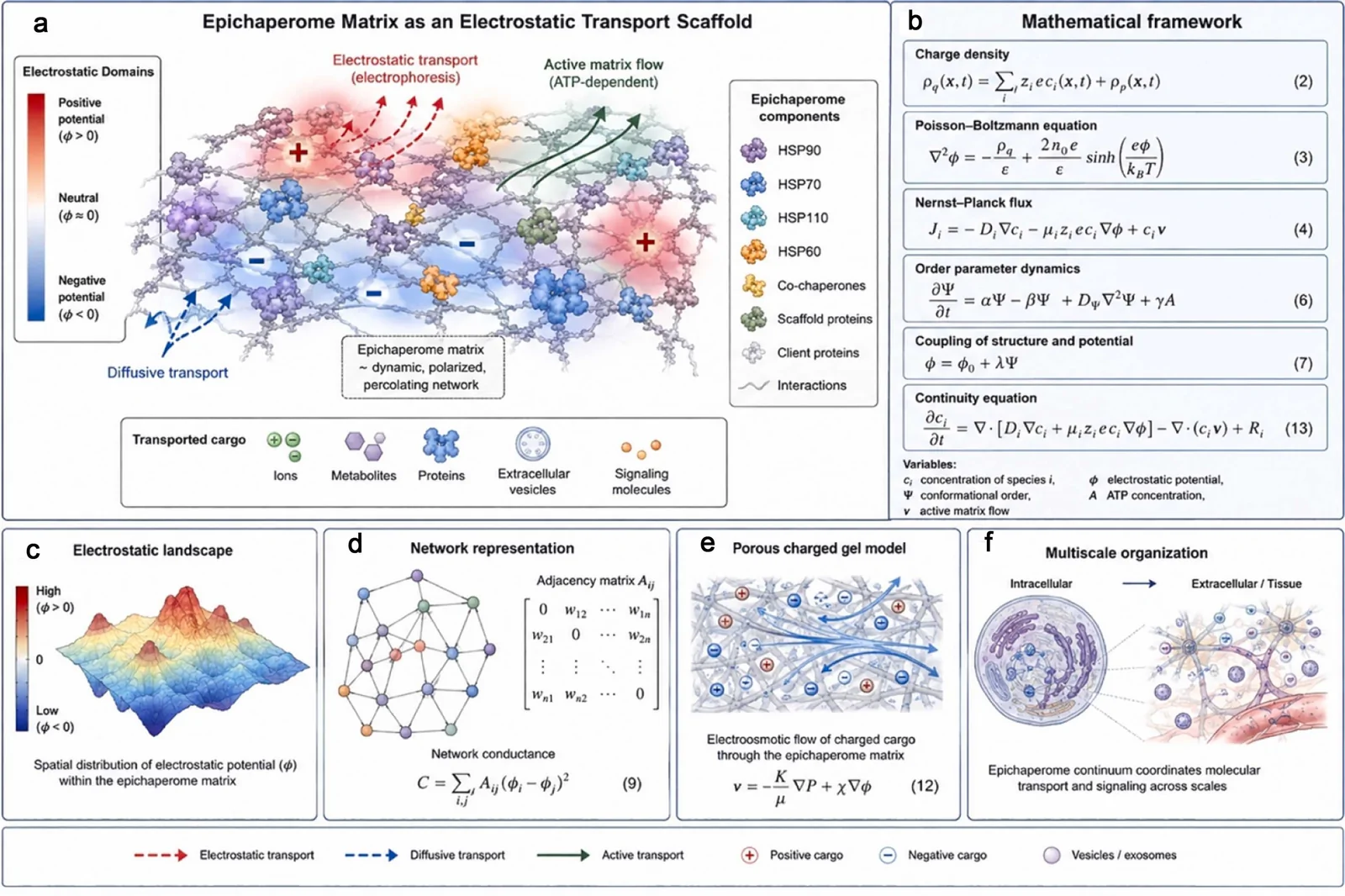
*Epichaperome Matrix Theory: a hypothetical electroactive and dynamically organized molecular scaffold.* (a) Conceptual representation of the epichaperome as a dynamic, electrically polarized, percolating molecular matrix composed of HSP90, HSP70, HSP110, HSP60, co-chaperones, scaffold proteins, and client proteins, generating heterogeneous electrostatic domains and potentially supporting diffusive, electrostatic, and ATP-dependent active transport of ions, metabolites, proteins, extracellular vesicles, and signaling molecules. (b) Mathematical framework describing charge density, electrostatic potential, molecular flux, conformational dynamics, coupling between matrix organization and electrostatic potential, and molecular continuity. (c-e) Complementary representations of the electrostatic landscape, interaction-network connectivity, and porous charged-gel behavior of the proposed matrix, illustrating how spatial polarization, network conductance, permeability, and electroosmotic coupling could influence molecular redistribution. (f) Multiscale extension of the model in which an epichaperome continuum could theoretically coordinate molecular organization, transport, and signaling from the intracellular environment to extracellular and tissue compartments.

A plausible theoretical framework for an epichaperome-based transport matrix can be constructed by treating the epichaperome not merely as a protein-protein interaction network but as a dynamic, electrically polarized, percolating biomolecular scaffold that generates spatial electrostatic asymmetry and thereby supports selective transport of ions, metabolites, proteins, extracellular vesicles, and signaling molecules (Figure 1).

Such a model is necessarily hypothetical because no experimental evidence currently demonstrates that epichaperomes function as a global transport system analogous to membranes or vascular networks. Nevertheless, as a systems biology and biophysical model, it can provide a useful conceptual framework for understanding how large-scale chaperome assemblies might regulate molecular flux within cells and tissues.

In this model, the epichaperome is considered a continuous nonequilibrium network composed primarily of interconnected molecular chaperones, including HSP90, HSP70, HSP110, HSP60, co-chaperones, scaffold proteins, kinases, phosphatases, and client proteins.^8^ Under stress conditions such as cancer, neurodegeneration, or chronic inflammation, these proteins assemble into stable supramolecular structures that extend far beyond conventional transient chaperone interactions. The resulting network behaves as a mesoscale matrix possessing emergent physical properties not present in individual components.

The central assumption of the model is that epichaperome assemblies generate local charge asymmetry through three mechanisms: unequal distribution of charged amino acid residues, ATP-dependent conformational cycling, and accumulation of phosphorylated client proteins. Because many epichaperome-associated proteins contain extensive acidic domains rich in glutamate and aspartate residues, as well as phosphorylated serine, threonine, and tyrosine residues, large regions of the network acquire significant negative electrostatic potential. Simultaneously, positively charged domains of HSP90, HSP70, DNA-binding proteins, RNA-binding proteins, and signaling molecules create localized positive potential wells. The resulting structure resembles a heterogeneous electrostatic landscape.

Let the spatial density of epichaperome components be represented by:

(1) $$\rho_{e}(x,t)$$

where x denotes spatial position and t time.

The local charge density can then be defined as:

(2) $$\rho_{q}\left(x,t\right)=\sum_{i}z_{i}ec_{i}\left(x,t\right)+\rho_{p}(x,t)$$

where $z_{i}$ is the valence of molecular species *i*, *e* is the elementary charge, *c*_*i*_ represents the local concentration of charged proteins, and *ρ*_*p*_ describes the charge contributed by phosphorylation and post-translational modifications.

The electrostatic potential field generated by the epichaperome satisfies the nonlinear Poisson-Boltzmann equation:

(3) $$\nabla^{2}\phi =-\frac{\rho_{q}}{\varepsilon }+\frac{2n_{0}e}{\varepsilon }\sinh\left(\frac{e\phi }{k_{B}T}\right)$$

where

ϕ is electrostatic potential,

ε is dielectric permittivity,

n_0_ is ionic strength,

$k_{B}$ is Boltzmann's constant,

and T is temperature.

This equation generates spatial electrostatic asymmetry throughout the epichaperome matrix.

Transport of molecules through the matrix can then be modeled using the Nernst-Planck formalism.^20^, ^21^ The flux of molecular species *i* is

(4) $$J_{i}=-D_{i}{\nabla c}_{i}-\mu_{i}z_{i}{\mathrm{ec}}_{i}\nabla \phi +c_{i}v$$

where

*D*_*i*_ is diffusion coefficient,

*μ*_*1*_ is electrophoretic mobility,

and v represents active matrix flow generated by ATP-dependent conformational dynamics.

The first term describes diffusion. The second term describes electrostatic transport along epichaperome-generated electric fields. The third term describes active transport resulting from collective molecular rearrangements.

A key feature of the model is the incorporation of ATP-driven conformational oscillations. Epichaperome proteins constantly cycle between structural states. Let the conformational order parameter be

(5) Ψ(x,t)

with dynamics:

(6) $$\frac{\partial \Psi }{\partial t}=\;\alpha \Psi -\beta \Psi +D_{\Psi }\nabla^{2}\Psi +\gamma A$$

where

A represents ATP concentration.

This equation resembles a Ginzburg-Landau system describing phase transitions. Regions with high ATP availability become structurally ordered and create coherent transport pathways.

The electrostatic potential becomes coupled to conformational state:

(7) $$\phi =\phi_{0}+\lambda \Psi$$

meaning that structural ordering directly modifies local electric fields.

To describe large-scale connectivity, the epichaperome can be modeled as a weighted graph where vertices represent proteins and edges represent stable interactions.

The adjacency matrix is

(8) $$A_{\mathrm{ij}}=\left\{\begin{array}{c} w_{\mathrm{ij},\mathrm{interaction}\;\mathrm{exists}}\\ 0,\mathrm{otherwise} \end{array}\right)$$

The transport efficiency between nodes is governed by network conductance^22^

(9) $$C=\sum_{i,j}A_{\mathrm{ij}}{\left(\phi_{i}-\phi_{j}\right)}^{2}$$

which is mathematically analogous to electrical current propagation through a resistor network.

In cancer-associated epichaperomes, increasing network connectivity produces higher conductance and more efficient molecular redistribution. A further extension treats the epichaperome as a porous charged gel.

Let porosity be

(10) η(x)

and permeability

(11) K(x)

Transport of large molecules such as signaling proteins, RNA complexes, or extracellular vesicles can then be described by a modified Darcy equation^23^, ^24^:

(12) $$v=-\frac{K}{\mu }\nabla P+\chi \nabla \phi$$

Where *P* is effective pressure,

*µ* is viscosity

and *χ* is electroosmotic coupling

This formulation predicts that charged macromolecules move through the epichaperome not solely by diffusion but also through electroosmotic flows generated by electrostatic gradients.

The complete transport equation becomes

(13) $$\frac{{\partial c}_{i}}{\partial t}=\nabla *\left[D_{i}\nabla c_{i}+\mu_{i}z_{i}ec_{i}\nabla \phi \right]-\nabla *\left(c_{i}v\right)+R_{i}$$

where

*R*_*i*_ represents biochemical reactions such as binding, release, phosphorylation, degradation, or client maturation.

An especially intriguing aspect of the model emerges when considering epichaperome phase behavior. Experimental evidence indicates that epichaperomes exhibit properties reminiscent of biomolecular condensates. The free-energy functional can therefore be expressed as:

(14) $$F=\int [{a\Psi }^{2}+{b\Psi }^{4}+k{\left(\nabla \Psi \right)}^{2}+\rho_{q}\phi ]\mathrm{dV}$$

Minimization of this free energy predicts spontaneous formation of highly ordered electrostatic domains. These domains function as transport channels analogous to conductive pathways in semiconductors.

One may further define an effective transport coefficient:

(15) $$T_{\mathrm{eff}}=D+\frac{\mu \mathrm{ze}\phi }{k_{B}T}+\Lambda \Psi$$

where

Λ describes structural enhancement of mobility.

Transport becomes maximal in highly ordered regions possessing strong electrostatic polarization.

At the whole-cell level, the epichaperome matrix can be conceptualized as a three-dimensional electroactive scaffold occupying much of the cytoplasmic volume. Rather than molecules diffusing randomly through cytosol, they experience guidance from epichaperome-generated electric fields and dynamic structural corridors. Negatively charged molecules may migrate toward electropositive domains, while positively charged proteins accumulate near acidic chaperone-rich regions. ATP-driven conformational waves continuously reorganize these pathways, creating a self-adapting transport system.

Extending the concept to tissues and blood, one can imagine an interconnected extracellular epichaperome network in which circulating epichaperome complexes form a distributed electrostatic matrix. In such a speculative model, transport of cytokines, metabolites, exosomes, and signaling proteins would be governed by coupled reaction-diffusion-electrostatic equations operating across multiple spatial scales.

The resulting theoretical framework resembles a hybrid of:

1. Membrane electrostatics (Poisson-Boltzmann theory),
2. Electrophoretic transport (Nernst-Planck equations),
3. Active matter physics,
4. Percolation network theory,
5. Biomolecular condensate thermodynamics,
6. Porous charged hydrogel transport models.

The overarching hypothesis is that the epichaperome behaves as a nonequilibrium electrostatically polarized matrix whose emergent charge asymmetry generates directed transport pathways. Such a system would not replace classical membrane transport or vascular circulation but could act as a superimposed organizational layer coordinating molecular distribution, signaling efficiency, and intracellular communication. This framework is mathematically self-consistent and biologically plausible as a systems-level model, although substantial experimental validation would be required to determine whether real epichaperomes possess transport properties approaching those predicted by the theory.

## The transcellular epichaperome continuum hypothesis

**Fig. 2:**
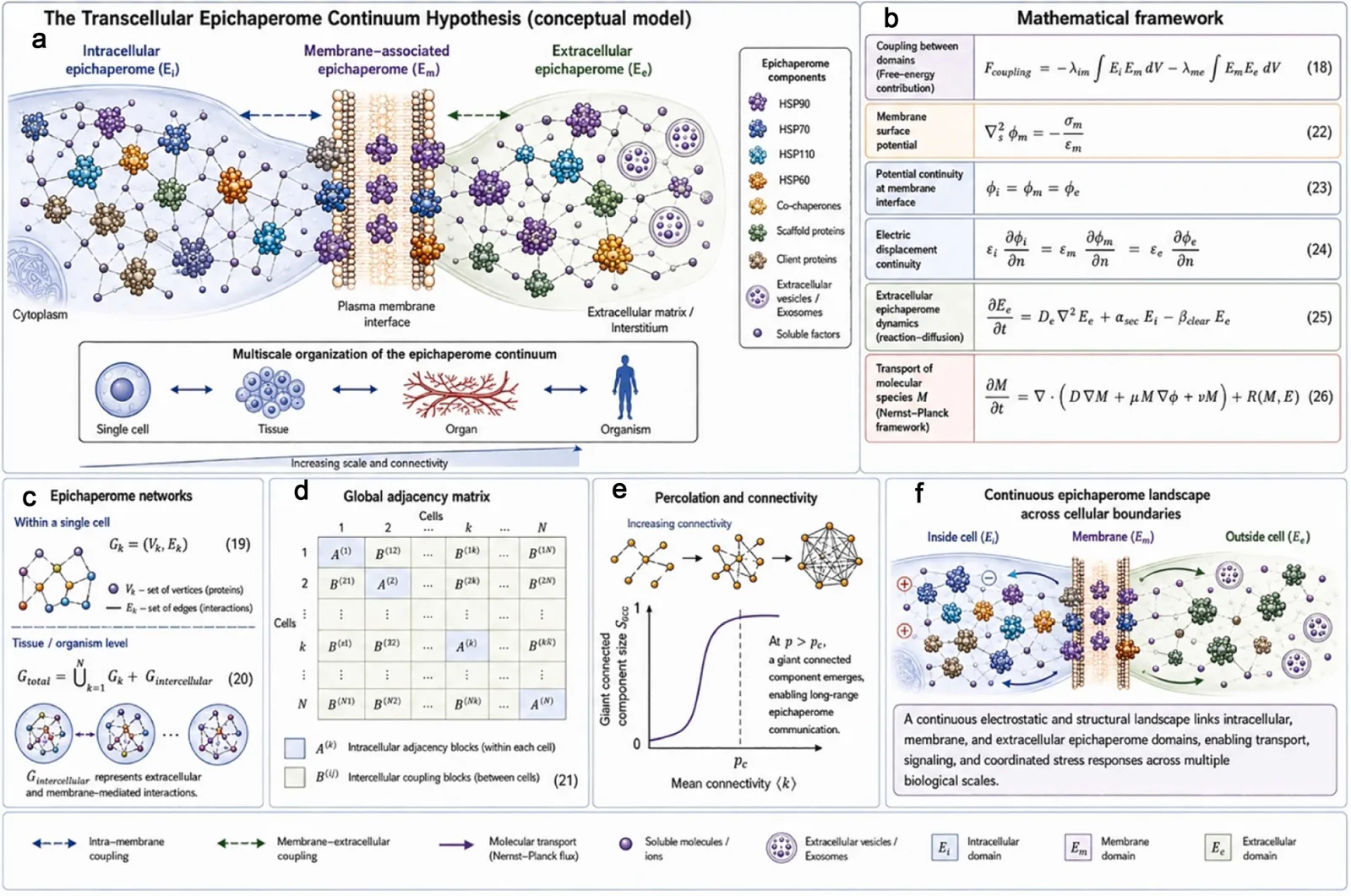
The Transcellular Epichaperome Continuum Hypothesis: a multiscale conceptual and mathematical framework. (a) Proposed continuum linking intracellular (E_*i*_), membrane-associated (E_*m*_), and extracellular (E_*e*_) epichaperome domains across cellular to organismal scales. (b) Mathematical framework describing interdomain coupling, membrane electrostatics, potential and displacement continuity, extracellular reaction-diffusion dynamics, and molecular transport. (c-d) Graph-theoretical and adjacency-matrix representations of intracellular and intercellular epichaperome connectivity. (e) Percolation-dependent network integration, illustrating the emergence of a giant connected component above the critical connectivity threshold (p_*c*_). (f) Integrated electrostatic and structural landscape linking intracellular, membrane-associated, and extracellular domains and potentially coordinating molecular transport, signaling, and adaptive responses across multiple spatial scales.

The EMT can be extended beyond the boundaries of individual cells by proposing that intracellular epichaperomes are physically and functionally coupled to plasma membrane-associated epichaperome assemblies,^13^, ^25^ which in turn interact with extracellular epichaperome structures, creating a multiscale transcellular continuum. In this extended framework, the epichaperome is no longer considered solely as an intracellular stress-associated protein network but rather as a hierarchical organizational system spanning intracellular, membrane, extracellular, tissue, and potentially organismal scales (Figure 2). Such a model suggests that molecular transport, signaling, and information processing occur not only through classical mechanisms such as diffusion, membrane transporters, cytoskeletal trafficking, extracellular vesicles, and vascular circulation, but also through an additional layer of organization generated by interconnected epichaperome structures.

The total epichaperome system may be described as a superposition of three interacting domains: intracellular epichaperome *E*_*i*_, membrane-associated epichaperome *E*_*m*_, and extracellular epichaperome *E*_*e*_. The total epichaperome density field is therefore represented as:

(16) $$E\;\left(x,t\right)=E_{i}\left(x,t\right)+E_{m}\left(x,t\right)+E_{e}(x,t)$$

where x denotes spatial coordinates and t represents time. The membrane-associated epichaperome acts as a coupling layer linking intracellular and extracellular compartments. The energetic state of the system can be described through a free-energy functional composed of intracellular, membrane, extracellular, and coupling contributions:

(17) $$F=F_{i}+F_{m}+F_{e}+F_{\mathrm{coupling}}$$

with

(18) $$F_{\mathrm{coupling}}=-\lambda_{\mathrm{im}}\int E_{i}E_{m}\mathrm{dV}-\lambda_{\mathrm{me}}\int E_{m}E_{e}\mathrm{dV}$$

where $\lambda_{\mathrm{im}}$ and $\lambda_{\mathrm{me}}$ characterize coupling strengths between neighboring domains. When these coupling coefficients become sufficiently large, the three epichaperome compartments behave as an integrated system rather than independent structures.

Within tissues, each cell contains its own epichaperome network represented as a graph where vertices correspond to proteins and edges represent stable interactions.

(19) $$G_{k}=(V_{k},A_{k})$$

where

$G_{k}$ the epichaperome network graph for cell,

*k* the index (identifier) of the specific cell,

$V_{k}$ the set of vertices (nodes) in the graph, representing proteins,

and $A_{k}$ represents the set of edges (arcs) in the graph, representing stable protein-protein interactions.

Tissue-level organization emerges when intracellular networks become coupled through extracellular epichaperome interactions. The resulting organismal graph can be represented as:

(20) $$G_{\mathrm{total}}=\overset{N}{\underset{k=1}{⋃}}G_{k}+G_{\mathrm{intercellular}}\;$$

where G_*intercellular*_ represents extracellular and membrane-mediated interactions. The adjacency matrix of the complete system contains intracellular blocks and intercellular coupling matrices:

(21) $$A=\left(\begin{array}{cccc} A_{1} & C_{12} & C_{13} & \ldots \\ C_{21} & A_{2} & C_{23} & \ldots \\ C_{31} & C_{32} & A_{3} & \ldots \\ \vdots & \vdots & \vdots & \ddots \end{array}\right)$$

The existence of an organism-wide epichaperome continuum depends upon percolation phenomena.^26^ As network connectivity increases beyond a critical threshold p_c_, a giant connected component emerges. Above this threshold, epichaperome-mediated communication pathways can theoretically span entire tissues and organ systems, allowing coordinated responses to stress across large biological distances.

The plasma membrane occupies a central position in this model because it serves as the interface through which intracellular and extracellular epichaperome systems communicate. Let the membrane-associated epichaperome density be described by a surface density function σm(r,t). The electrostatic potential generated on the membrane surface satisfies

(22) $$\nabla_{s}^{2}\phi_{m}=-\frac{\sigma_{m}}{\varepsilon_{m}}$$

where $\nabla_{s}^{2}$ is the surface Laplacian operator and ε_m_ is the effective dielectric permittivity of the membrane-associated epichaperome layer. Continuity conditions require that intracellular, membrane, and extracellular potentials remain coupled:

(23) $$\phi_{i}=\phi_{m}=\phi_{e}$$

at the membrane interface. Similarly, electric displacement continuity requires

(24) $$\varepsilon_{i}\frac{{\partial \phi }_{i}}{\partial n}=\varepsilon_{m}\frac{{\partial \phi }_{m}}{\partial n}=\varepsilon_{e}\frac{{\partial \phi }_{e}}{\partial n}$$

thus generating a continuous electrostatic landscape extending across cellular boundaries. Consequently, electrostatic gradients generated within intracellular epichaperome assemblies can influence extracellular regions and vice versa.

The extracellular compartment is modeled as a dynamic epichaperome field generated through secretion, extracellular vesicle release, and extracellular stabilization of chaperone complexes. The extracellular epichaperome density obeys a reaction-diffusion equation:

(25) $$\frac{\partial E_{e}}{\partial t}=D_{e}\nabla^{2}E_{e}+\alpha_{\sec}E_{i}-\beta_{\mathrm{clear}}E_{e}$$

where $D_{e}$ is the extracellular diffusion coefficient, $\alpha_{\sec}$ represents secretion rates from cells, and $\beta_{\mathrm{clear}}$ characterizes extracellular degradation and clearance. Secreted HSP90, HSP70, co-chaperones, extracellular vesicles, exosomes, and stress-induced protein condensates collectively contribute to the formation of a distributed extracellular epichaperome matrix.

Transport of molecular species throughout the integrated epichaperome continuum can be described using a generalized Nernst-Planck framework. For a molecular species M, the transport equation becomes

(26) $$\frac{\partial M}{\partial t}=\nabla *\left(D\nabla M+\mu M\nabla \phi +\mathrm{vM}\right)+R(M,E)$$

where *D* is the diffusion coefficient, *μ* is electrophoretic mobility, ϕ is the total electrostatic potential, v represents active transport generated by ATP-dependent epichaperome dynamics, and R(M,E) describes biochemical reactions including binding, release, phosphorylation, degradation, and client maturation. In this formulation,

(27) $$\phi =\phi_{i}+\phi_{m}+\phi_{e}$$

represents the unified electrostatic field generated by intracellular, membrane-associated, and extracellular epichaperome domains. Consequently, transport pathways are no longer confined to individual cellular compartments but may theoretically extend across multiple cells.

At larger scales, the epichaperome continuum may be treated as an anisotropic conductive medium. A conductivity tensor

(28) $$\sum =\left(\begin{array}{ccc} \sigma_{\mathrm{xx}} & \sigma_{\mathrm{xy}} & \sigma_{\mathrm{xz}}\\ \sigma_{\mathrm{yx}} & \sigma_{\mathrm{yy}} & \sigma_{\mathrm{yz}}\\ \sigma_{\mathrm{zx}} & \sigma_{\mathrm{xy}} & \sigma_{\mathrm{zz}} \end{array}\right)$$

characterizes directional transport properties of the tissue-scale epichaperome network. The resulting flux obeys

(29) J=−∑∇ϕ

analogous to electrical current propagation through conductive materials. Regions possessing dense epichaperome connectivity exhibit enhanced conductance and therefore facilitate efficient redistribution of signaling molecules and metabolites.

Extracellular vesicles introduce an additional dynamic component into the network. Let *V(x,t)* represent vesicle density. Their behavior may be described by

(30) $$\frac{\partial V}{\partial t}=D_{v}\nabla^{2}V+\gamma E_{e}-\delta V$$

where $D_{v}$ is vesicle diffusivity, γ is the vesicle generation rate, and δ describes vesicle removal. Vesicles transport proteins, RNAs, metabolites, and signaling molecules while simultaneously functioning as mobile epichaperome nodes. The effective connectivity between cells therefore becomes

(31) $$C_{\mathrm{ij}}=C_{\mathrm{static}}+C_{\mathrm{vesicle}}$$

combining static extracellular interactions with mobile vesicle-mediated coupling. This process greatly enhances long-range network integration.

An important feature of the model arises from ATP-dependent conformational dynamics. Individual cellular epichaperomes continuously undergo structural oscillations driven by ATP hydrolysis. If neighboring cells become coupled through extracellular epichaperome interactions, these oscillations may synchronize. Such synchronization can be described using a Kuramoto-type model^27^, ^28^:

(32) $$\frac{d\theta_{i}}{\mathrm{dt}}=\omega_{i}+K\sum_{j}\sin\left(\theta_{j}-\theta_{i}\right)$$

where $\theta_{i}$ represents the conformational phase of the epichaperome in cell *i, ω*_*i*_ is its intrinsic frequency, and *K* is the coupling constant. Above a critical coupling threshold, cells exhibit collective synchronization, generating coordinated transport pathways across tissues.

At the organismal level, the intracellular, membrane, and extracellular epichaperome compartments may be unified into a single field variable

(33) $$\Psi \left(x,t\right)=E_{i}+E_{m}+E_{e}$$

representing the total epichaperome density throughout the organism. The evolution of this field may be described by

(34) $$\frac{\partial \Psi }{\partial t}=D_{\Psi }\nabla^{2}\Psi +\alpha \Psi -\beta \Psi^{3}+\gamma {\left|\nabla \phi \right|}^{2}$$

which resembles reaction-diffusion systems known to generate self-organizing biological structures. Solutions to this equation predict the spontaneous formation of transport corridors, signaling domains, stress-adaptation zones, and long-range communication pathways within tissues.

In its fully developed form, this theoretical framework proposes that intracellular epichaperomes, membrane-bound epichaperomes, and extracellular epichaperome assemblies form a hierarchical, nonequilibrium transport continuum extending across biological scales. The plasma membrane functions as the critical coupling interface that links intracellular and extracellular epichaperome domains, while extracellular vesicles and secreted chaperone complexes extend connectivity between neighboring cells. The resulting system can be viewed as a dynamic electroactive matrix superimposed upon classical mechanisms of diffusion, membrane transport, extracellular vesicle trafficking, and vascular circulation. Within this framework, molecular transport becomes influenced not only by concentration gradients and biochemical interactions but also by emergent electrostatic, structural, and network properties of the epichaperome continuum. The central prediction is that once network connectivity exceeds a critical percolation threshold, tissues may exhibit coordinated transcellular transport and signaling behaviors arising from the collective dynamics of interconnected epichaperome networks. Although highly speculative and currently unsupported by direct experimental evidence, such a model provides a mathematically consistent systems-level framework for exploring how stress-induced chaperome assemblies might contribute to biological organization across scales ranging from individual cells to the entire organism.

## Discussion

**Fig. 3:**
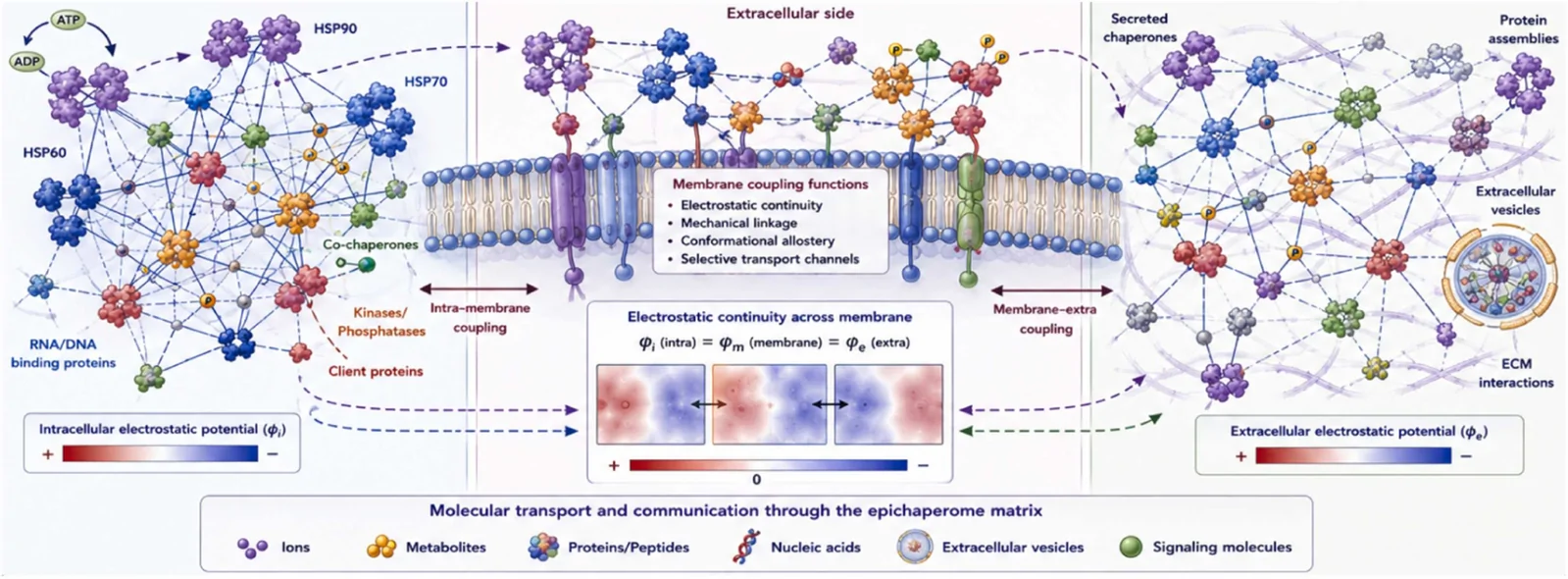
Schematic presentation of epichaperome matrix.

The present work extends the emerging concept of the epichaperome from a stress-induced protein interaction network to a theoretical systems-level framework in which epichaperomes function as dynamic organizational matrices capable of influencing molecular transport, signaling, and biological integration across multiple spatial scales. While experimental studies have established that epichaperomes arise through extensive remodeling of chaperone interactions during chronic stress and disease, the potential physical consequences of these highly interconnected structures remain largely unexplored. The EMT and its extension into a transcellular epichaperome continuum provide a conceptual framework for investigating how large-scale chaperome assemblies may contribute to the organization of molecular flux within cells, between cells, and potentially throughout tissues and organs (Figure 3).

The central premise of this model is that epichaperomes possess emergent properties that are not predictable from the behavior of individual molecular chaperones. This perspective is consistent with a growing body of evidence indicating that biological function increasingly emerges at mesoscale levels of organization. Biomolecular condensates, membrane nanodomains, cytoskeletal networks, extracellular matrices, and signaling platforms all demonstrate that collective molecular assemblies frequently exhibit physical behaviors distinct from those of their constituent components. Epichaperomes may represent another example of such emergent biological organization. The remarkable increase in connectivity observed in disease-associated epichaperomes suggests that they may function not merely as protein stabilization complexes but as integrated regulatory structures capable of coordinating multiple cellular processes simultaneously.

One of the most intriguing aspects of the proposed model is the emergence of electrostatic organization as a potential mechanism for long-range coordination. Proteins are inherently charged macromolecules whose interactions are strongly influenced by local electrostatic environments. Epichaperomes contain large numbers of phosphorylated signaling proteins, ATP-binding proteins, acidic and basic domains, nucleic acid-binding proteins, and molecular chaperones undergoing continuous conformational cycling. Collectively, these features create the possibility that epichaperome assemblies generate heterogeneous electrostatic landscapes capable of influencing molecular movement. Although the electrostatic fields produced by individual proteins are generally short-ranged in physiological ionic environments, highly organized and densely interconnected protein assemblies may exhibit collective electrostatic behaviors extending beyond those expected from isolated molecules. Similar principles have been proposed for cytoskeletal structures, membrane interfaces, and biomolecular condensates. The present model suggests that epichaperomes may constitute another class of biologically relevant electroactive structures.

The spatial scale of the proposed effects should be considered explicitly. At the molecular and mesoscale levels, electrostatic interactions could influence proteins, ions, and other charged molecules within or near epichaperome assemblies, potentially extending across tens to hundreds of nanometers. At the cellular and tissue levels, direct electrostatic effects would be strongly limited by ionic screening; longer-range coordination would therefore more likely depend on network connectivity, active remodeling, vesicular transport, and cytoskeletal or cell-cell coupling. Accordingly, the proposed transcellular effects should be understood as propagation of local interactions through interconnected networks rather than as direct electrostatic fields extending across macroscopic distances.

The application of Poisson-Boltzmann-type descriptions to epichaperome assemblies requires several simplifying assumptions. In the highly crowded intracellular environment, electrostatic interactions are strongly affected by ionic strength, counterion condensation, molecular crowding, dielectric heterogeneity, and the finite size and complex geometry of proteins. Consequently, classical Poisson-Boltzmann formulations should be regarded here as a continuum-level approximation for estimating effective electrostatic potentials rather than as an exact representation of molecular-scale interactions within densely packed epichaperomes. In particular, physiological ionic screening is expected to substantially limit the range of direct electrostatic interactions. Future extensions of the model should therefore incorporate spatially heterogeneous dielectric properties, explicit ion effects, molecular crowding, and, where appropriate, nonlinear or modified Poisson-Boltzmann formulations. These considerations define important boundaries for interpreting the predicted electrostatic effects and provide experimentally testable directions for refining the theory.

The incorporation of ATP-dependent conformational dynamics further distinguishes the epichaperome continuum from static structural frameworks. Chaperones are fundamentally nonequilibrium machines whose activity is driven by continuous ATP consumption.^29^, ^30^ Consequently, epichaperomes should not be viewed as passive scaffolds but rather as active matter systems capable of generating dynamic structural fluctuations. Theoretical coupling between ATP-dependent conformational oscillations and transport processes introduces the possibility that epichaperomes may continuously remodel transport pathways in response to changing physiological conditions. Such adaptive behavior would be consistent with the well-established role of molecular chaperones in facilitating cellular adaptation to stress. In this context, transport regulation becomes an emergent consequence of stress-induced network reorganization rather than a dedicated transport mechanism evolved specifically for cargo movement.

The proposed transcellular extension of the model introduces a particularly provocative concept: that plasma membrane-associated epichaperomes may couple intracellular and extracellular chaperome networks into larger organizational units. Several observations provide indirect support for considering such a possibility. Numerous molecular chaperones, including HSP90, HSP70, HSP60, and co-chaperones, have been detected at the plasma membrane and within extracellular environments.^25^, ^31^, ^32^ Extracellular chaperones participate in immune signaling,^33^, ^34^ tissue remodeling, wound healing,^35^ cancer progression,^36^ and inflammatory responses.^37^ Furthermore, extracellular vesicles, exosomes, and membrane-derived particles frequently contain chaperones and chaperone-associated proteins.^38^, ^39^ These findings indicate that chaperome components are not restricted to intracellular compartments but can participate in intercellular communication processes. The present theory proposes that these observations may represent components of a broader organizational system linking intracellular and extracellular molecular environments.

Importantly, the model does not require the existence of continuous protein filaments physically spanning multiple cells. Instead, connectivity emerges through a combination of membrane-associated assemblies, extracellular protein complexes, vesicle-mediated interactions, electrostatic coupling, and dynamic network reorganization. Such an arrangement resembles other biological systems in which distributed interactions generate coordinated behavior without requiring direct structural continuity. Examples include neural networks, endocrine signaling systems, immune communication networks, and extracellular matrix-mediated mechanotransduction. In the proposed framework, the epichaperome continuum acts as an additional layer of biological organization superimposed upon these established communication pathways.

The percolation-based component of the theory offers a potentially useful framework for understanding stress-dependent transitions in cellular organization. Epichaperomes are known to become particularly abundant in pathological states characterized by chronic stress, including cancer, neurodegenerative disease, and persistent inflammation. Percolation theory predicts that complex networks often undergo abrupt transitions once connectivity exceeds critical thresholds. Below such thresholds, interactions remain local and fragmented. Above them, large-scale coordinated behavior emerges through the formation of giant connected components. Applied to epichaperome biology, this concept suggests that disease-associated epichaperomes may not simply represent quantitatively larger chaperone networks but qualitatively different organizational states exhibiting fundamentally new emergent properties. Such transitions could potentially explain why epichaperome formation is frequently associated with major alterations in cellular behavior, signaling integration, and stress adaptation.^7^

The model also provides an interesting conceptual bridge between epichaperome biology and the rapidly expanding field of biomolecular phase separation. Increasing evidence suggests that many cellular processes are organized through dynamic condensates formed by multivalent interactions among proteins and nucleic acids.^40^, ^41^ Epichaperomes share several characteristics with phase-organized systems, including high molecular connectivity, stress-dependent assembly, altered physical properties, and the capacity to concentrate specific molecular populations. The free-energy formulations introduced in the present framework suggest that epichaperomes may generate highly ordered domains capable of facilitating preferential transport and signaling. Future studies investigating the relationship between epichaperomes and biomolecular condensates may therefore provide important insights into the physical basis of epichaperome organization.

Despite its conceptual appeal, the proposed framework remains highly speculative and faces several significant challenges. The greatest limitation is the absence of direct experimental evidence demonstrating that epichaperomes participate in transport processes beyond their established roles in proteostasis and signaling regulation. However, it should be noted that several observations from the molecular chaperone field provide indirect support for the possibility that chaperone assemblies may contribute to membrane-associated transport and trafficking processes. In particular, studies by Nimmervoll et al demonstrated that HSP70 can oligomerize on the plasma membrane and participate in endocytic events, suggesting that chaperone complexes are capable of organizing into higher-order structures at cellular membranes and influencing membrane dynamics.^42^ These findings indicate that molecular chaperones are not confined exclusively to intracellular protein-folding functions but can also operate at the cell surface where they participate in processes involving membrane remodeling, vesicular trafficking, and cellular uptake mechanisms. While the mathematical formalism presented here is internally consistent, mathematical consistency alone does not establish biological validity. Numerous assumptions underlying the model remain untested, including the existence of large-scale electrostatic asymmetry generated by epichaperomes, the formation of stable membrane-associated epichaperome interfaces, the presence of organized extracellular epichaperome matrices, and the capacity of these structures to influence molecular transport over biologically meaningful distances.

An important consideration for future development of the theory is the distinction between experimentally supported components of epichaperome biology and more speculative extensions of the present framework. The existence of intracellular chaperone and co-chaperone interaction networks, stress-dependent epichaperome formation, ATP-dependent chaperone dynamics, and membrane-associated chaperone populations is relatively well established and therefore provides the strongest biological foundation for the model. In contrast, the proposed electrostatic transport properties, long-range coupling between epichaperome domains, extracellular epichaperome organization, and a transcellular epichaperome continuum remain largely theoretical and require independent experimental validation. Several potentially important variables are also not explicitly incorporated into the present formulation, including local ion concentrations and ionic strength, membrane lipid composition and fluidity, cytoskeletal architecture, molecular crowding, extracellular matrix properties, vesicle-mediated transport, mechanical forces, and cell-to-cell heterogeneity. Incorporating these variables in future iterations may improve the biological realism of the model and allow its predictions to be tested across increasingly complex cellular and tissue environments. Thus, the current framework should be viewed as a theoretical foundation that builds upon relatively consolidated chaperome biology while deliberately extending into less explored physical and extracellular dimensions that remain open to experimental investigation.

Several experimentally testable predictions arise directly from the proposed theory, with some being particularly accessible to near-term investigation. Initial studies could determine whether membrane-associated epichaperomes exhibit non-random spatial organization, whether molecular mobility is altered within epichaperome-rich regions, and whether these assemblies possess measurable electrostatic or mechanical properties. A further critical test would be to determine whether perturbation of epichaperome architecture alters molecular transport independently of conventional transport mechanisms. More complex studies could then examine whether extracellular chaperome assemblies display structural connectivity and whether advanced imaging can detect coordinated intracellular and extracellular epichaperome dynamics across neighboring cells. Together, these experiments would provide a stepwise and experimentally tractable strategy for testing the core assumptions of the framework before extending validation to more complex transcellular and tissue-level predictions. Confirmation of these predictions would provide increasing support for the proposed theory and help define which of its predicted emergent properties are biologically realized.

The theory also suggests new perspectives on disease biology. Cancer, neurodegenerative disorders, chronic inflammatory diseases, and aging are all characterized by profound remodeling of chaperone networks. If epichaperomes contribute to molecular transport and signaling organization, pathological alterations in epichaperome architecture could influence not only proteostasis but also the broader spatial organization of cellular communication. Disease progression might therefore involve the emergence of aberrant organizational fields that coordinate pathological signaling across tissues. Such a possibility remains speculative but could help explain the extensive systems-level effects associated with chronic stress states.

## Conclusion

In conclusion, the EMT and the transcellular epichaperome continuum hypothesis propose a fundamentally expanded view of epichaperome biology. Rather than functioning solely as stress-induced protein interaction networks, epichaperomes are envisioned as dynamic, nonequilibrium organizational matrices capable of integrating molecular transport, signaling, and adaptive responses across multiple biological scales. The plasma membrane emerges as a critical interface linking intracellular and extracellular epichaperome domains, while extracellular chaperones and vesicle-mediated interactions provide mechanisms for intercellular connectivity. Although substantial experimental validation will be required to determine whether biological systems possess the properties predicted by this model, the framework provides a new theoretical perspective for investigating how stress-induced molecular assemblies may contribute to higher-order biological organization. By integrating concepts from stress biology, biophysics, network theory, active matter physics, and systems biology, this model establishes a foundation for future studies exploring the possibility that epichaperomes represent not only regulators of proteostasis but also fundamental organizers of biological complexity.

## Funding and support

The research was supported by the Technical University of Munich (TUM) within the DFG funding program Open Access Publishing.

## CRediT authorship contribution statement

**Maxim Shevtsov:** Writing – review & editing, Writing – original draft, Project administration, Methodology, Investigation, Funding acquisition, Conceptualization.

## Declarations of interest

The authors declare that they have no known competing financial interests or personal relationships that could have appeared to influence the work reported in this paper.

## Data Availability

Data will be made available on request.
